# 

**Published:** 1880-09

**Authors:** 


### CONTENTS:

ART. I.—Anæsthesia by Ethyl Bromide. By H. Augustus Wilson, M. D. .......... 193
ART. II.—Diagnosis of Malignant Tumors of the Upper Jaw in Youth. By L. Mc Lane Tiffany, M. D. .......... 206
ART. III.—Robert Arthur, M. D., D. D. S. By W. W. H. Thackston, D. D. S. ..... 214
ART. IV.—History of Diploma Selling .......... 220
ART. V.—The Administration of Chloroform. By Rich. L. Macdonnell, M. D. ..... 225

### EDITORIAL, ETC.

What Others Say .......... 233

Advances in Medical Education .......... 235

### BIBLIOGRAPHICAL.

Homœopathy .......... 237

### MONTHLY SUMMARY.

Chancres of the Tonsil and Mouth .......... 239
Effects of Smoking on the Heart .......... 240

